# The Infant-Food Problem

**Published:** 1888-06

**Authors:** Wm. B. Atkinson

**Affiliations:** Professor of Diseases of Children and Sanitary Science in the Medico-Chirurgical College, Philadelphia.


ARTICLE VI.

### THE INFANT-FOOD PROBLEM.

BY WM. B. ATKINSON, A. M.; M. D.,

[Professor of Diseases of Children and Sanitary Science in the Medico-Chirurgical College, Philadelphia.]

[Read in the Section on Diseases of Children, at the Thirty-Ninth Meeting of the American Medical Association, May 10, 1888.

To the general practitioner everywhere, there constantly comes the question, What means shall be employed to prevent the terrible mortality among infants deprived of their natural food, the mother's breast-milk? As it is in very many cases impossible to place the child outside the walls of a large city, this want of proper hygienic surroundings acts as one great factor in the production of disease. But perhaps the most active cause of disease is the exhaustion of the vital powers from the want of those articles which, being properly and readily assimilated, aid to maintain the body in its highest and healthiest condition. We all know that, other things being equal, that child which has been able to keep its system in the best state, its blood rich and pure, its muscles plump and firm, is sure to pass through an epidemic of children's affections either entirely unscathed or suffering only from a slight attack, readily throwing off the disease and never being troubled with the sequelæ.

Defective nutrition, then, is the predominant factor in the causation of the fearful mortality everywhere observed among children. We need only point to the statistics of children's hospitals, foundling asylums and similar institutions to show the truth of this proposition.

To us, as physicians and sanitarians, as citizens earnest for the welfare of this great Republic, this comes with powerful import. An additional fact also appeals to us when we learn that the vast majority of these are offspring of the native born, while those who survive are largely the children of foreigners. This is shown by the valuable statistics of such investigators as Dr. Nathan Allen. Though we are compelled to admit that other causes, and one a very potent factor, produce the great disproportion between offspring of natives and foreigners, yet it must be admitted that the truth of our original proposition still is evident, that defective vitality causes a vast majority of deaths among infants, and even in children of larger growth.

The latter fact is constantly shown by the great mortality which prevails when, by reason of short crops or other causes, the people are unable to procure the food needed to maintain their systems up to par and thus resist the inroads of disease.

It goes without saying that the infant should be raised on its mother's milk whenever possible. When, for any cause, this fails, then comes the question, what shall be the substitute? Abroad the milk of asses and goats is in quite common use. Cow's milk, being that most easily obtained, is most largely employed in this country. This being the fact, we next come to the consideration as to how the two kinds of milk differ, and what is needed in order to cause that of the cow most nearly to approach that of the human being.

Cow's milk contains more proteid matter, more fat, more mineral matter and less sugar. and as a rule, in health human milk is alkaline, while cow's milk is often slightly acid. One special difficulty with cow's milk is that its casein is more or less likely to form an insoluble mass by contact with the gastric juice, while the casein of human milk is in part a peptone, and forms a very delicate coagulum when in contact with the gastric juice.

The effort is always to produce a food for infants closely resembling in its composition the mother's milk, and the nearer this is reached in all its details, the more surely will such food prove wholesome and valuable to the infant.

Our idea of a standard infant food, then, would be produced as follows: Be sure to obtain the milk of a healthy cow. Just here we may premise that we do not believe in the common fallacy, "one cow's milk." The mixture of the milk of several healthy cows is more sure to give an article of real value. Undoubtedly, many in this audience can substantiate the claim that it is most usually the pet cow, from which is obtained the milk which is put up for the sick baby, that receives all the banging, hurrying and pelting, and, as we all know, is thus likely to yield a milk which may actually be poisonous in its nature. The best combination would be pure milk diluted with sufficient pure water to reduce the relative proportion of albuminoids and mineral constituents most nearly to that of human milk, then partially peptonize or digest it, and finally add a soluble carbo-hydrate with sufficient alkali to produce as close a resemblance to breast-milk as may be. We must not forget that peptonizing milk does not relieve us of the need of being sure that the milk is at the outset pure and fresh.

The milk supply of large cities has now become one of the great problems of the day. Churned in the cars to the city, then more thoroughly churned in the wagons over the wretchedly paved streets, distributed in many cases from doubtful cans by persons of much more doubtful appearance as to their own cleanliness; the flavor often aided by the puffing of a cigar or filthy pipe on the part of the distributor, the article is received in many cases in a receptacle of equal doubt as to cleanliness, is is placed, perhaps, in a food chest or so-called refrigerator, exposed to the atmospheric contact of other articles of food. Is it to be wondered at that the milk becomes of a very doubtful form as to its propriety as an infant aliment?

To a certain extent, these objections are met by the new plan of delivering what is called "whole milk." The milk is very carefully placed in glass jars immediately after being drawn from the cow, these, being quite full, are hermetically sealed so that there can be no opportunity of churning or adulteration, or the absorption of odors or disease germs. For children who have passed the age of infancy, I have long been in the habit of urging the employment, particularly during hot weather, of what is called "evaporated milk." Its claims were that it was the milk from healthy, well-fed cows, and, being of a density greater than cream, churning and souring were less likely to occur during its transition to the city. Again, it was very much less ready to absorb or appropriate the odors, etc., to which it might be subjected. I have found this more easily borne by the child, and repeatedly I have been compelled to substitute it for the "condensed milk," where a certain proportion of sugar is added in order to preserve the article.

For these reasons, Prof. Vaughan urges the use of dried milk solids, that is, they can be transported without injury from any distance, and, if properly prepared, may be kept without putrefaction occurring. Now, if such pure milk from perfectly healthy cows were partially predigested by the process of peptonisation with fresh pancreatine, and to this dextrine added, thus supplying the carbo-hydrate, we would then come as near the production of a proper food for infants as might be possible in the absence of the breast-milk.

By recent researches, we have been taught that dextrine is the best form of carbo-hydrate, as it is non-fermentable and does not irritate the stomach of the infant, is easily assimilated, and, unlike cane sugar or maltose, is not likely to take on acid fermentation. Roasted wheat flour has long been employed and recommended as an article of food for infants, and particularly where diarrhœa is present. The reason of this is because this process converts the starch of the flour into dextrine.

The malt sugar or so-called "Liebig foods" are no doubt often valuable, particularly in infantile constipation, for their laxative effects, but are extremely liable to continue a diarrhœa or increase it. When these are used for their laxative effect, it is safer to use them alone rather than with milk, lest their fermentative tendency be aggravated by the presence of too great a quantity of albuminoid matter.

I am incited to this remark by the remembrance that the "Liebig foods" do not of themselves meet the requirements demanded for infantile nutrition, unless with the addition of cow's milk. By an examination of the analyses of such mixtures, we find that they add no essential to cow's milk; nor do these foods act chemically upon the casein, nor physically by reason of their solubility; and, as I have before remarked, they may give rise to disorders of digestion in consequence of the readiness with which they take on fermentation.

Farinaceous foods are of course out of the question, because of the absence of ptyalin in the secretion of the salivary glands in the earlier years of infancy. The addition of starchy matters to cow's milk with the purpose of rendering the coagulum less dense and more easily broken up in the stomach, as has been recommended by some authorities, is wrong in principle; it really adds an indigestible element, which cannot fail to act as a foreign body sure to produce fermentation, acidity, diarrhœa and the usual train of evils.

The milk foods, when diluted with water in accordance with directions, should correspond in nutritive value with human milk. Now, that this correspondence should be more nearly perfect, they should also be partially predigested or peptonized, in order that the casein may be rendered more acceptable. It is also necessary that sugar in some form should be added.

In peptonizing milk, it is of the greatest importance that the pancreatic extract which is employed should be pure and fresh. The odor of some digestive ferments as furnished by the stores is such as to give rise to the suspicion that they are already assuming the putrefactive tendency. In fact, it is a very difficult matter to preserve them, as it is well known that the products of the pancreas are much more readily decomposed than any known animal substance. Hence the greatest care will be necessary that there shall not be the slightest possibility of the presence of the presence of putrefactive germs in any of these articles than may be employed to aid in the preparation of the diet for infants. The peptonizing of milk, although apparently a very simple matter as practiced in the laboratory. yet is scarcely feasible in the household.

Another point is of great importance. Malt sugar is eminently prone to absorb mixture, and hence it should not be combined with dried milk and then put in bottles or other forms of package for family use, because, as these packages are only partially used at one time, the balance is extremely liable to absorb moisture, resulting in fermentation, and this is more especially the case in hot weather or when kept in a hot room.

We cannot too strongly urge upon all who are compelled to prepare food for infants the great, the imperative necessity of using only water that has been boiled. To the medical man, the reason is plain, yet it would not be amiss for him to explain in each instance why this should be done. Just here it is equally important to see that the water is not cooled by the addition of ice, as we may thus return at once to the water the very organisms which the boiling was intended to expel. I am impelled to this remark by the remembrance of an inspection, just made for the State Board of Health of Pennsylvania. The subject of complaint was the ponds from which the ice was obtained to supply the demands of a large town. These ponds were filled with water from a stream really nothing but a drain for a full gradeyard, one or more slaughter-houses, a large number of cesspools which were in contact use, and a large area of swamp land.

In diluting any form of infant food we should give positive, definitive quantities. Undoubtedly all of us have encountered many instances where the child was really starving, while apparently receiving a large quantity of fluid. The fact is that the dilution had been carried too far.

It is unnecessary for me to occupy your time with further points as to times for feeding, nor of necessity for using bottles, etc.

Before closing, I may remark that, in my investigation of foods for the preparation of a paper which may be read elsewhere, I have received from my friend Chief Medical Purveyor Baxter, of the United States Army, a tabulated analysis of some fifteen forms of foods. Of these, only four contained more than 10 per cent. of nutritive material, thus showing that even here we are likely to be deceived, and to be employing an article as useless for its proposed purpose as the too largely diluted food of the infant already mentioned.

In conclusion, permit me to say that it has long been my custom, not only in my practice, but also in my teachings to urge the giving of less medicine, using it only when imperatively demanded, and to insist upon the value of proper hygiene and proper nourishment, believing that these alone, in many cases, will at once place the child on the road to health, and if persevered in will, as a rule, maintain it there.—*Jour. Am. Med. Association.*

